# Review of the Interaction Between Body Composition and Clinical Outcomes in Metastatic Renal Cell Cancer Treated With Targeted Therapies

**DOI:** 10.15586/jkcvhl.2016.45

**Published:** 2016-03-22

**Authors:** Steven M. Yip, Daniel Y.C. Heng, Patricia A. Tang

**Affiliations:** Department of Oncology, University of Calgary, Tom Baker Cancer Centre, Calgary, Alberta, Canada.

**Keywords:** adiposity, body composition, obesity, renal cell carcinoma, sarcopenia, targeted therapy, toxicity

## Abstract

Treatment of metastatic renal cell cancer (mRCC) currently focuses on inhibition of the vascular endothelial growth factor pathway and the mammalian target of rapamycin (mTOR) pathway. Obesity confers a higher risk of RCC. However, the influence of obesity on clinical outcomes in mRCC in the era of targeted therapy is less clear. This review focuses on the impact of body composition on targeted therapy outcomes in mRCC. The International Metastatic Renal Cell Carcinoma Database Consortium database has the largest series of patients evaluating the impact of body mass index (BMI) on outcomes in mRCC patients treated with targeted therapy. Overall survival was significantly improved in overweight patients (BMI ≥ 25 kg/m^2^), and this observation was externally validated in patients who participated in Pfizer trials. In contrast, sarcopenia is consistently associated with increased toxicity to inhibitors of angiogenesis and mTOR. Strengthening patients with mRCC and sarcopenia, through a structured exercise program and dietary intervention, may improve outcomes in mRCC treated with targeted therapies. At the same time, the paradox of obesity being a risk factor for RCC while offering a better overall survival in response to targeted therapy needs to be further evaluated.

## Introduction

Treatment of metastatic renal cell cancer (mRCC) currently focuses on inhibition of the vascular endothelial growth factor (VEGF) pathway and the mammalian target of rapamycin (mTOR) pathway. Although predictive biomarkers for targeted therapy have yet to be validated, there are prognostic models that can stratify mRCC patients into low-, intermediate-, and high-risk groups. Two commonly used models include the International Metastatic Renal Cell Carcinoma Database Consortium (IMDC) model (1) and the Memorial Sloan-Kettering Cancer Center (MSKCC) criteria (2, 3) (**Table 1**).

**Table 1.** Comparison of multivariable prognostic factor models in metastatic renal cell carcinoma

Model	MSKCC	IMDC criteria
	Motzer et al. (2, 3)	Heng et al. (1)
Patient population	463 patients treated with interferon alpha on prospective clinical trials	645 patients treated with sunitinib, sorafenib, or bevacizumab at multiple North American centers
Prognostic factors	KPS < 80%	KPS < 80%
	LDH > 1.5 × ULN	Corrected calcium > ULN
	Corrected calcium > 10 mg/dL	Hemoglobin < LLN
	(2.5 mmol/L)	Disease-free interval < 1 year
	Hemoglobin < LLN	Neutrophils > ULN
	Disease-free interval < 1 year	Platelets > ULN
Favorable risk	No risk factors, mOS 30 months	No risk factors, mOS not reached
Intermediate risk	1 risk factor, mOS 14 months	1–2 risk factors, mOS 27 months
Poor risk	2–3 risk factors, mOS 5 months	3 or more risk factors, mOS 8.8 months

IMDC: International Metastatic Renal Cell Carcinoma Database Consortium; KPS: Karnofsky performance status; LDH: Lactate dehydrogenase; LLN: Lower limit of normal; mOS: median overall survival; MSKCC: Memorial Sloan-Kettering Cancer Center; ULN: Upper limit of normal.

Obesity confers a higher risk of RCC (4–7); however, the influence of obesity on clinical outcomes in mRCC in the era of targeted therapy is less clear. The World Health Organization utilizes body mass index (BMI; weight divided by height squared) to define the terms “overweight” (BMI 25.0–29.9 kg/m^2^) and “obesity” (BMI ≥ 30 kg/m^2^) (8). However, BMI does not accurately reflect body composition, the proportion of lean tissue to fat; nor does BMI account for sarcopenia, the loss of skeletal muscle tissue. Computed tomography (CT) is often used to assess response to therapy as part of routine care. Cross-sectional imaging can be utilized to quantify skeletal muscle density (SMD) and adipose tissue. In addition, relative distribution of fat can be localized to the visceral or subcutaneous compartments (visceral fat area [VFA] and superficial fat area [SFA]).

Higher BMI may negatively influence outcomes through commonly associated comorbidities of diabetes and cardiovascular disease (9). It may alter drug concentrations and pharmacokinetics of targeted therapies that are dosed independent of weight. Obesity may activate oncogenic pathways and create an inflammatory state. This is postulated to occur via elevations in interleukins (IL-6, IL-1β, and IL-1 receptor antagonist), tumor necrosis factor, and C-reactive protein (10). Furthermore, a proangiogenic state is created by the production of factors such as VEGF and leptin by adipose tissue (11). An obese body composition also can promote and activate the mTOR pathway through reactive oxygen species (12), as well as elevated levels of insulin and insulin-like growth factor (13).

Obesity is paradoxically associated with better prognosis, particularly in the setting of nephrectomized patients with RCC (14, 15). **Table 2** summarizes the findings of studies, which examine this relationship between survival rates in mRCC and body composition metrics. This review paper will focus on the impact of body composition on targeted therapy outcomes in mRCC.

**Table 2.** Retrospective studies evaluating the impact of body composition on outcome in metastatic renal cell carcinoma patients treated with targeted therapies

Study population	Body composition cutpoint for obesity	Impact on clinical outcomes
Choueiri et al. 475 North American patients included in the IMDC database (16)	BMI ≥ 30 kg/m^2^	High BMI associated with improved OS
		HR 0.67
		(95% CI 0.49–0.91, p = 0.01)
Albiges et al. 1,975 patients (17)	BMI ≥ 25 kg/m^2^	High BMI associated with improved median OS (25.6 vs 17.1 months, p < 0.0001)
Albiges et al. 4,657 patients from Pfizer trials (18)	BMI ≥ 25 kg/m^2^	High BMI: improved OS (HR 0.830, p = 0.0008, 95% CI 0.743–0.925)

BMI: body mass index; BSA: body surface area; HR: hazard ratio; OS: overall survival; CI: confidence interval.

## Literature search strategy

A PubMed and Medline literature search was performed for the time period 1994 to 2015 with the following search terms:

sarcopen^*^, BMI, body mass, cachexia, BSA, body surface area, body composition, renal cell ca^*^, RCC, kidney cancer, prognos^*^, outcome^*^, response, predict^*^, mTOR, everolimus, sirolimus, sunitinib, PD1, PDL1. Additionally, American Society of Clinical Oncology meeting proceedings were searched with the following search terms: BMI, body, BSA, renal cell. Articles in any language were included, and all levels of evidence were considered. The retrieved articles’ relevant references were also reviewed for possible inclusion. Eleven articles (eight published, three abstracts) evaluated body composition as a prognostic factor for targeted therapy outcomes in mRCC.

## Impact of BMI on outcomes

Body composition and its potential influence on targeted therapy outcomes were initially assessed in a retrospective study of 475 mRCC patients treated with antiangiogenic therapy. Choueiri et al. (16) identified that obesity (BMI > 30 kg/m^2^) was independently associated with greater overall survival (OS) (hazard ratio [HR] 0.67, 95% confidence interval [CI] 0.49–0.91, p = 0.01), after adjusting for the IMDC model criteria. The IMDC updated this analysis in a larger dataset of 1,975 patients treated with targeted therapy (17). Overweight or obese patients (BMI ≥ 25 kg/m^2^) had a significantly longer median OS compared with underweight or normal patients (25.6 vs 17.1 months, p < 0.0001), which remained significant after adjusting for IMDC model criteria.

This finding was externally validated in a cohort of 4,657 mRCC patients on phase II and III Pfizer trials from 2003 to 2013 (18). Overweight or obese patients (BMI ≥ 25 kg/m^2^) had a longer OS, in comparison with the low BMI group (BMI < 25 kg/m^2^) (23.4 vs 14.5 months, HR 0.830, 95% CI 0.743–0.925, p = 0.0008), while controlling for the IMDC prognostic risk criteria. Similarly, high BMI was associated with improved progression-free survival (PFS) (HR 0.821, 95% CI 0.746–0.903, p < 0.0001) and response rate (odds ratio 1.527, 95% CI 1.258–1.855, p < 0.001), in contrast to low BMI. Interestingly, the results were similar when stratified by line of therapy. The favorable outcome associated with elevated BMI was observed only in clear cell mRCC, when stratified by histology.

There was question of whether tolerability of therapy in higher BMI patients played a role in producing these findings. However, in the IMDC dataset, rates of early discontinuation due to adverse events did not differ between the two BMI groups, and therefore this was unlikely a cause of bias (17). Additionally, the toxicity patterns were similar in the high- and low-BMI groups in the external Pfizer validation set (18).

The biologic rationale for the association between BMI and outcomes is not clear. Fatty acid synthase (FASN) is a key enzyme involved in the production of fatty acids. FASN has emerged as a metabolic oncogene with an important role in tumor growth and survival (19). There was a trend to improved OS in the elevated BMI group (p = 0.07) in the Cancer Genome Atlas clear cell mRCC dataset (n = 61) (18). High BMI was associated with low FASN gene expression (p = 0.034), and FASN expression (using the median as a cutpoint) was inversely associated with OS (p = 0.002). *FASN* gene was evaluated using immunohistochemistry (IHC) in the IMDC biospecimen cohort (17). Median OS was significantly improved in FASN IHC negative compared with positive patients (27.5 vs 14.5 months, p = 0.005). FASN metabolism may contribute to the development of mRCC and therefore may represent a novel therapeutic target. These results are hypothesis-generating.

## Volumetric assessment of body composition

BMI is a relatively crude measurement of body composition. Other groups have evaluated more granular metrics of body composition such as volumetric assessment of fat and muscle (**Table 3**). Software programs can be used to identify the visceral and subcutaneous visceral adipose compartments using Houndsfield units at specific landmarks on CT, followed by calculation of the cross-sectional area. In contrast to the IMDC data, BMI was not prognostic in other smaller retrospective studies that incorporate these volumetric assessments (20–22). Steffens et al. (20) evaluated the impact of baseline BMI, body surface area (BSA, m^2^), VFA, and SFA in 116 mRCC patients. VFA and SFA were calculated with ImageJ software using the umbilicus as the landmark. The cutoffs used were a BMI ≥ 30 kg/m^2^, a BSA above the European average > 1.74 for women and BSA > 1.98 for men, and finally an SFA or a VFA above the median of the patient cohort. On multivariate Cox regression analysis incorporating histological subtype and MSKCC status, there was no significant association between PFS and OS with elevated BMI and BSA. Elevated SFA and VFA were independently associated with improved OS and PFS (SFA: HR 3.41, 95% CI 1.61–7.25, p = 0.001; VFA: HR 2.97, 95% CI 1.36–6.47, p = 0.006).

**Table 3.** Influence of volumetric assessments of body composition on clinical outcomes in RCC

Study	Body composition cutpoint	Impact of body composition on outcomes			
		Elevated body mass index (BMI)	Elevated superficial fat area (SFA)	Elevated visceral fat area (VAT)	Sarcopenia or skeletal muscle density (SMD)
Steffens et al. 116 European patients (20). VFA and SFA were computed with ImageJ software at the level of the umbilicus	BMI ≥ 30 kg/m^2^, BSA above the European average > 1.74 for women and BSA > 1.98 for men. SFA or VFA above the median of the patient cohort	Not prognostic	High SFA, improved OS. HR 3.41 (95% CI 1.61–7.25, p = 0.001)	High VFA, improved OS. HR 2.97 (95% CI 1.36–6.47, p = 0.006)	N/A
Ladoire et al. 64 European patients (21). Baseline VFA and SFA were calculated with CT at the level of the umbilicus and calculated with ImageJ software	BMI > 30 kg/m^2^, SFA > median, VFA > median	Not prognostic	High SFA not prognostic	High VFA associated with shorter OS in patients treated with VEGF inhibitors. HR 6.26 (95% CI 2.29–17.08, p < 0.001)	N/A
Gu et al. 124 Chinese patients (22). SAT and VAT measured with CT at L3, and calculated with ImageJ software	High VAT 33.3 cm^2^/m^2^	Not prognostic	SAT associated with improved OS. HR 0.987 (95% CI 0.974–1.000, p = 0.048)	High VAT associated with improved OS. HR 0.981 (95% CI 0.969–0.993, p = 0.002)	Not prognostic
**Studies Focused on Sarcopenia**					
Antoun et al. 149 European and Canadian patients (23). Skeletal mass density assessed by CT SliceOMatic software at L3	Sarcopenia: below the median for patients of the same sex within the study population	Not prognostic	SAT not prognostic	VAT not prognostic	Shorter OS (1/2) in patients of low SMD compared with high SMD (14 vs 29 months, p = 0.001). Low SMD OS. HR 1.9 (95% CI 1.3–2.9)
Antoun et al. 55 European and Canadian patients treated with sorafenib (24). CT: volumetric assessment at the L3 with SliceOMatic software	Sarcopenia: more than two standard deviations below average on volumetric assessment at L3	Mean BMI of patients with DLT significantly lower than patients who tolerated full dose (23.1 vs 26.0 kg/m^2^, p < 0.03)	N/A	N/A	Males with sarcopenia more likely to experience DLT compared with nonsarcopenic (37% vs 5%, p < 0.04)
McCabe et al. 112 European patients (26). Sarcopenia was assessed using Appendicular Skeletal Muscle Index (ASMI), measuring at the L3 landmark on CT	Sarcopenia defined as ASMI <7.26 kg/m^2^ for males and <5.45 kg/m^2^ for females	N/A	N/A	N/A	Sarcopenic patients more likely to experience severe treatment-related toxicity compared with nonsarcopenic (Pearson chi-square value 12.82, p = 0.001)
Huillard et al. 61 European patients receiving Sunitinib (27). Sarcopenia was assessed using L3 landmark on CT, using software ImageJ software v1.42q	Sarcopenia defined using the L3 landmark on CT, with sex-specific cutoff values of 55.4 cm^2^/m^2^ for males and 38.9 cm^2^/m^2^ for females	Not prognostic	N/A	N/A	Sarcopenics with BMI < 25 more likely to experience DLT compared with nonsarcopenics with BMI > 25 (50% vs 19.5%, p = 0.01)
Cushen et al. 55 European patients receiving sunitinib (28). Skeletal muscle mass and sarcopenia assessed using Osiris image software and measured with CT images that extended from L3	Sarcopenia cutoffs 55.4 cm^2^/m^2^ for males and 38.9 cm^2^/m^2^ for females	Not prognostic	N/A	N/A	Patients with SMM < 25th percentile experienced more DLT compared with those with SMM > 75th percentile (92% vs 57%, p = 0.05). Sarcopenia was not predictive for early DLTs

L3: the level of the third lumbar vertebra; BMI: body mass index; BSA: body surface area; CI: confidence interval; DLT: dose-limiting toxicity; HR: hazard ratio; N/A: not available; OS: overall survival; RCC: renal cell cancer; SAT: subcutaneous adiposity tissue; SFA: superficial fat area; SMD: skeletal muscle density; VAT: visceral adiposity tissue.

Ladoire et al. (21) evaluated the prognostic impact of BMI, SFA, and VFA in French patients with mRCC using similar methods as Steffens et al. The median was used to dichotomize SFA and VFA values into high versus low. High VFA was associated with significantly shorter time to progression and OS (HR 6.26, 95% CI 2.29–17.08, p < 0.001) in patients treated with antiangiogenic drugs (n = 64) but not in patients treated with cytokines (n = 49), on multivariate analysis, including MSKCC group. An obese BMI (> 30 kg/m^2^) and high SFA were not prognostic. Ladoire et al. suggested that high VFA was a predictive factor since it was associated with worse outcomes for patients treated with antiangiogenic therapy but not cytokines. These results differ from the larger IMDC dataset, in which overweight or obese patients (BMI ≥ 25 kg/m^2^) had a significantly longer median OS compared with underweight or normal patients (17).

Gu et al. (22) retrospectively assessed BMI, BSA, visceral adipose tissue (VAT) index, subcutaneous adipose tissue (SAT), and SMD in mRCC patients treated with VEGF and mTOR therapies (n = 124). SAT and VAT were measured with CT at the level of the third lumbar vertebra and calculated with ImageJ software. Based on Cox regression modeling adjusting for age, sex, and IMDC criteria, both VAT and SAT indices were significantly associated with prolonged OS (VAT: HR 0.981; 95% CI 0.969–0.993, p = 0.002; SAT: HR 0.987, 95% CI: 0.974–1.000, p = 0.048). However, no significant association was found between OS and BMI (p = 0.121), nor BSA (p = 0.335). Sarcopenia, or a depletion of skeletal muscle, can occur independent of adiposity. SMD had no significant association with OS (HR 1.000, 95% CI 0.986–1.013, p = 0.950).

Antoun et al. (23) evaluated the impact of CT assessed body composition in a subset of patients from mRCC trials treated with sorafenib, sunitinib, everolimus, and placebo. When adjusted for the IMDC model score, mRCC patients with high SMD had significantly longer OS (HR 1.85; p = 0.02) and PFS (HR 1.81; p = 0.02), in contrast to patients with low SMD. Antoun et al. incorporated SMD into the IMDC model score and created a new model. Median OS for patients with a favorable-risk IMDC model score and high SMD was 35 months (95% CI 24–43 months); 22 months (95% CI 14–27 months) for patients with an intermediate-risk IMDC model score and high SMD as well as a favorable-risk IMDC model score and low SMD; and 8 months (95% CI 6–12 months) for patients with an intermediate-risk IMDC model score and low SMD. This model has not been externally validated. No relationship was found between BMI and outcomes. SAT and VAT were not prognostic.

## The impact of sarcopenia on adverse events from targeted therapy

Sarcopenia was evaluated in a subset of mRCC patients from the TARGET trial (sorafenib vs placebo after progression on standard therapy) (24). Sarcopenia was present in 72% of patients with a BMI < 25 and 34% of obese patients. Treatment with sorafenib was associated with a significant decrease in skeletal muscle in comparison with placebo (8.0% loss, p < 0.01). Preclinical models suggest that the skeletal muscle loss associated with sorafenib may be mediated by downstream effects of mTOR inhibition (25). Frequency of sorafenib-induced dose-limiting toxicities was highest in sarcopenic patients whose BMI < 25 kg/m^2^, and lowest in nonsarcopenic patients who were overweight or obese (p = 0.03) (24). These results suggest that sarcopenia in mRCC is a predictor of sorafenib-induced toxicity. Since sorafenib promotes muscle loss severe adverse events may be more frequent in sarcopenic patients. A future area of research would be to individualize the dose of a targeted therapy based on a patient’s skeletal muscle mass, in order to decrease dose-limiting toxicities and optimize clinical outcomes (23).

A similar interaction between sarcopenia and toxicity was observed in a retrospective analysis of 112 mRCC patients treated with mTOR inhibitors, immunotherapy, VEGF inhibitors, Tyrosine Kinase Inhibitors (TKIs), and best supportive care (26). The prevalence of sarcopenia was 20.5% at baseline and increased to 38.4% at the end of the evaluation. Sarcopenia was independently associated with increased frequency of severe (common toxicity criteria grade > 2) treatment toxicity (Pearson chi-square value 12.82; p = 0.001).

Huillard et al. (27) explored the association between sarcopenia and toxicities in 61 mRCC patients treated with sunitinib. Sarcopenia was present in 32 patients (52.5%), and 20 sarcopenic patients (32.8%) also had a BMI < 25 kg/m^2^. Patients with sarcopenia and BMI<25 kg/m^2^ experienced significantly more dose-limiting toxicities (DLTs) (OR = 4.1, 95% CI 1.3–13.3, p = 0.01). During the first cycle, they also experienced more grade 3 toxicities (p = 0.04), as well as more cumulative grade 2 or 3 toxicities (p = 0.008). Sunitinib was permanently discontinued during the first cycle in 30% of sarcopenic patients, as compared with 2.4% of the remaining patients (p = 0.01). On multivariate analysis, the combination of sarcopenia and BMI<25 kg/m^2^ was the only independent predictor of early DLTs (p = 0.04). However, the presence of sarcopenia had no significant impact on OS (p = 0.75) and PFS (p = 0.071).

Cushen et al. (28) investigated the impact of fat-free mass and skeletal muscle mass (SMM; metabolic tissues such as the liver and kidney, intracellular and extracellular water, and bone) on DLTs in mRCC patients treated with sunitinib. Sarcopenia was present in 33% of the patients (18/55). DLTs were inversely associated with SMM; 92% of the patients with SMM < 25th percentile experienced DLTs, in contrast to 57% of those patients with SMM > 75th percentile (p = 0.05). Patients with low fat-free mass (n = 4) experienced significantly more DLTs than those with high fat-free mass (n = 2, p = 0.002), but it is unclear what cutoff was used to determine the differentiation between these two groups.

## Discussion

The IMDC database has the largest series of patients evaluating the impact of BMI on outcomes in mRCC patients treated with targeted therapy. OS was significantly improved in overweight patients (BMI ≥ 25 kg/m^2^), and this observation was externally validated in patients who participated in Pfizer trials (17, 18). *FASN* gene is an emerging oncogene in mRCC, and high BMI may be a surrogate for low FASN levels. If this finding is externally validated prospectively, future studies should optimize the FASN assay and determine whether inhibition of this pathway has the potential to improve outcomes for mRCC.

CT may provide a more refined description of body composition than relatively crude measurements such as BMI. The impact of adipose tissue on mRCC outcomes is unclear. High VFA and SFA were associated with improved OS in studies performed by Steffens et al. (20) and Gu et al. (22). Conversely, increased VFA was associated with worse outcomes were in Ladoire’s cohort (21). Antoun et al. (23) found no association between VFA or SFA and outcomes. Sarcopenia was associated with worse outcomes by Antoun et al. (23), but not in the cohort evaluated by Gu et al. (22). These four cohorts did not observe a significant association of BMI with outcomes, but were all smaller than the IMDC cohort (17). These investigators used different combinations of imaging software programs and anatomical landmarks. Further refinement of this technology is required, and the cutpoints for sarcopenia, VFA, and SFA require further validation in a larger cohort of patients. These studies also focused on baseline measurements of BMI, and SFA and VFA (17, 18, 20–23). Further studies evaluating the paradox of obesity being a risk factor as wells a prognostic marker of response to targeted therapy are needed.

Toxicity from targeted therapy appears to be independent of BMI (17, 18). Retrospective series evaluating sarcopenia consistently demonstrate a relationship between sarcopenia and increased toxicity from targeted agents (24, 26–28). These studies were small, and the ideal method for evaluating sarcopenia as well as the optimum cutoff point has yet to be established.

What is the impact of sarcopenia on mRCC patients who are treated with targeted therapy? Sarcopenia is consistently associated with increased toxicity to inhibitors of angiogenesis and mTOR. A structured exercise program and dietary intervention may strengthen patients with mRCC and improve response to targeted therapy. Prospective studies evaluating the impact of diet and exercise on targeted therapy tolerance, quality of life, and body composition are warranted.

Future longitudinal studies should evaluate the prognostic impact of sarcopenia, VAT, SFA, BMI, and BSA in mRCC patients treated with targeted therapy. This would require controlling for other prognostic variables in a very large patient cohort to fully address this issue. This may facilitate the development of more refined prognostic models of mRCC treated with targeted therapy.
